# At the X-Roads of Sex and Genetics in Pulmonary Arterial Hypertension

**DOI:** 10.3390/genes11111371

**Published:** 2020-11-20

**Authors:** Meghan M. Cirulis, Mark W. Dodson, Lynn M. Brown, Samuel M. Brown, Tim Lahm, Greg Elliott

**Affiliations:** 1 Division of Pulmonary, Critical Care and Occupational Medicine, University of Utah, Salt Lake City, UT 84132, USA; mark.dodson@imail.org (M.W.D.); Lynn.brown@imail.org (L.M.B.); Samuel.brown@imail.org (S.M.B.); Greg.Elliott_MD@imail.org (G.E.); 2 Division of Pulmonary and Critical Care Medicine, Intermountain Medical Center, Salt Lake City, UT 84107, USA; 3 Division of Pulmonary, Critical Care, Sleep and Occupational Medicine, Department of Medicine, Indiana University School of Medicine, Indianapolis, IN 46202, USA; tlahm@iu.edu; 4 Richard L. Roudebush Veterans Affairs Medical Center, Indianapolis, IN 46202, USA; 5 Department of Anatomy, Cell Biology & Physiology, Indiana University School of Medicine, Indianapolis, IN 46202, USA

**Keywords:** bone morphogenetic protein receptor type 2, heritable, familial, estrogen, estradiol, penetrance, gender, PAH

## Abstract

Group 1 pulmonary hypertension (pulmonary arterial hypertension; PAH) is a rare disease characterized by remodeling of the small pulmonary arteries leading to progressive elevation of pulmonary vascular resistance, ultimately leading to right ventricular failure and death. Deleterious mutations in the serine-threonine receptor bone morphogenetic protein receptor 2 (*BMPR2*; a central mediator of bone morphogenetic protein (BMP) signaling) and female sex are known risk factors for the development of PAH in humans. In this narrative review, we explore the complex interplay between the BMP and estrogen signaling pathways, and the potentially synergistic mechanisms by which these signaling cascades increase the risk of developing PAH. A comprehensive understanding of these tangled pathways may reveal therapeutic targets to prevent or slow the progression of PAH.

## 1. Introduction

Group 1 pulmonary hypertension (pulmonary arterial hypertension; PAH) is a rare disease characterized by remodeling of the small pulmonary arteries which leads to the progressive elevation of pulmonary vascular resistance (PVR), and ultimately to right ventricular failure and death. While numerous inciting factors are known (e.g., connective tissue disease, HIV infection, drug and toxin exposure), the pathobiology of PAH generally convenes on a final common pathway of endothelial (EC) and smooth muscle cell (SMC) dysfunction, with an imbalance of apoptotic and proliferative signaling, vasoconstriction, and structural changes in vessel walls [1]. Deficiency of bone morphogenetic protein receptor 2 (BMPR2), a serine threonine kinase “type II” receptor in the transforming growth factor (TGF)-β superfamily, has been inexorably identified as a central mediator in this process [2].

Heterozygous germline mutations in *BMPR2* were first associated with PAH via genetic linkage analysis of families with the disease [3,4]. Since this discovery in 2000, further analysis of up- and down-stream signaling through the receptor, including both canonical and non-canonical pathways, has illuminated several mechanisms by which deficiency in BMPR2 signaling leads to PAH [5]. Not only are deleterious mutations in *BMPR2* associated with both heritable (~80%) and idiopathic (~20%) PAH [6,7,8], but decreased BMPR2 expression and signaling has also been demonstrated in other subtypes of PAH and non-PAH pulmonary hypertension (PH) in the absence of mutations [9,10]. Furthermore, the presence of a deleterious *BMPR2* mutation in heritable, idiopathic, and anorexigen-associated PAH portends a more severe clinical phenotype and decreased survival [11,12,13,14]. Despite the strong association between *BMPR2* mutations and the development of PAH, and despite the high frequency of *BMPR2* mutations in heritable PAH, having a *BMPR2* mutation alone is not sufficient; heterozygous carriers of deleterious *BMPR2* mutations only have an approximately 20% lifetime risk of disease penetrance [15]. Decades of investigation have revealed that there are likely multiple genetic and environmental “second hits” that may be necessary to spur PAH development in the setting of a deleterious *BMPR2* mutation [2].

## 2. The “Estrogen Puzzle” of PAH

One piece of the complex pathobiology of PAH is biologic sex and the “estrogen puzzle”, as it is referred to in the literature. In various animal models, estrogen and estrogen metabolites have been shown to protect the organism from developing PH in the setting of other provoking factors, whereas in human registry studies, a striking female predominance suggests increased susceptibility to disease [16]. Female carriers of deleterious *BMPR2* variants are more likely to develop PAH compared to males; however, once diagnosed, women are less likely than men to have severe disease [17]. A recently published meta-analysis of clinical trials also suggests that men with *BMPR2* mutations have more severe disease, but interestingly more men with idiopathic or heritable PAH were found to have a pathogenic *BMPR2* variant [18].

Increasingly recognized sex differences in right ventricular (RV) adaptation to chronic PH contribute to the “estrogen puzzle” of PAH and likely play a significant role in disease severity and associated mortality. Studies of PH in several distinct rat models have demonstrated that at baseline female rats have better RV function than males [19], ovariectomy attenuates the beneficial effect of female sex [19], and that the restoration of estrogen signaling (genomic and non-genomic) prevents progression of and can rescue the failing RV phenotype in both male and female rats via alterations in metabolism, inflammation, collagen deposition/fibrosis, and angiogenesis [19,20,21,22,23,24]. Human studies support these findings in healthy subjects and those with PAH. In the MESA-RV study, higher estradiol (E2) levels in healthy post-menopausal women using hormone replacement therapy were associated with higher RV ejection fraction and lower RV end-systolic volume [25]. In idiopathic PAH at baseline, men have lower RV ejection fraction and stroke volume compared to age-matched females despite similar pulmonary artery pressure (PAP) and PVR [26]. Two independent investigations demonstrated that after initiation of PAH-specific therapy, only women show improvement in RV function despite similar improvement in PVR between the sexes [27,28]. Disparate RV recovery is thought to explain, at least in part, the poorer prognosis seen in men.

Despite strong evidence for a substantial role, female sex and the effects of estrogen signaling do not fully explain the observed sex differences in PAH. Other sex-driven differences in the hormonal milieu (e.g., testosterone, dehydroepiandrosterone (DHEA), and progesterone), as well as non-hormonal sex effects (e.g., the recent finding that a Y-chromosome-encoded transcription factor may mediate *BMPR2* expression [29,30]) likely contribute to the complex and differential effects of biologic sex on pulmonary vascular remodeling and RV adaptation [31].

In this review, we will explore one piece of the intricate “estrogen puzzle”, specifically how estrogen and estrogen metabolites interact with the BMPR2 signaling pathway. Readers are directed to two recent reviews for a more comprehensive overview of the interaction between sex and PAH [31,32].

## 3. *BMPR2* Signaling

BMPR2 is a type II constitutively active serine-threonine kinase receptor integral to canonical bone morphogenetic protein (BMP) signaling. BMPR2 signaling occurs in both the EC and SMC of the pulmonary vasculature. Signal transduction is activated by BMP binding and the formation of a heterotetrameric complex of two dimers of type I and type II receptors. Type I receptors include activin receptor-like kinases (ALKs) 1–7. Complexing of the two receptors allows for phosphorylation of the downstream substrate proteins: receptor-regulated Smads (R-Smads), specifically Smads 1, 5, and 8 in BMP signaling. Activated R-Smads then associate with a co-Smad (Smad4) and translocate to the nucleus where they bind to BMP response element DNA sequences and promote gene expression of transcription factors such as inhibitor of DNA binding factors (ID1, ID2 and ID3) [2]. The inhibitor of DNA binding (also known as inhibitor of differentiation) proteins are known to have important regulatory effects on vascular homeostasis [33]. BMPR2 also activates “non-canonical” signaling pathways such as extracellular signal-related kinase (ERK), p38 mitogen-activated protein kinase (MAPK), Lin11, Isl-1, Mec-3 domain kinase (LIMK), Wingless (Wnt pathway), and NOTCH. The complex regulation of both the canonical and non-canonical pathways occurs at multiple levels, including via co-receptors (endoglin), pseudoreceptors, BMP antagonists, and inhibitory Smad proteins [34]. In addition to mutations in *BMPR2* itself, mutations in a number of components of the BMPR2 signaling pathway are linked to the development of PAH, including *ALK1* [35], *SMAD8* [36], *BMP9* [37], and *CAV1* (encoding caveolin-1) [38]. Although acting at different points in the signaling cascade, all of these mutations cause a deficiency in BMPR2 signaling, which is ultimately thought to drive the vascular remodeling and dysregulation central to PAH pathogenesis [2].

## 4. Estrogen Signaling

Similar to the BMPR2 signaling cascade, essential components of estrogen signaling pathways are expressed in the ECs, vascular SMCs, and fibroblasts responsible for vascular remodeling and the development of PAH [39,40]. Three primary estrogens (estrone, E1; estradiol, E2; estriol, E3) and their metabolites signal through two classical estrogen receptors (ERα and ERβ) and one newly discovered G-protein-coupled receptor (GPER) [41]. In the absence of pregnancy, E2 is the most abundant estrogen. Estrogens primarily signal via “genomic” and “non-genomic” pathways; the former facilitating the classic role of estrogens as transcription factors in the nucleus, the latter triggering rapid effects such as ion channel, kinase, endothelial nitric oxide synthase (eNOS) and prostacyclin synthase activation in the cytoplasm [42,43,44]. Via 2-, 4-, or 16-hydroxylation, E1 and E2 are metabolized to active compounds with varying potency and activity, signaling through ER-dependent and independent mechanisms. The 2-hydroxylation metabolites are generally considered weakly or anti-mitogenic, anti-estrogenic, and do not signal through an ER [45]. For example, 2-methoxyestradiol (2-ME) and 2-hydroxyestradiol (2-OHE) are considered to be anti-proliferative. In ECs, 2-ME is a potent modulator of nitric oxide (NO), prostacyclin, and endothelin synthesis [45,46]. On the other hand, 16-hydroxylation produces 16α-OHE_1_, which is similar in potency to E2 and has potent pro-proliferative, pro-inflammatory, and pro-angiogenic effects [47,48].

## 5. Estrogen and *BMPR2*

**Figure 1:**
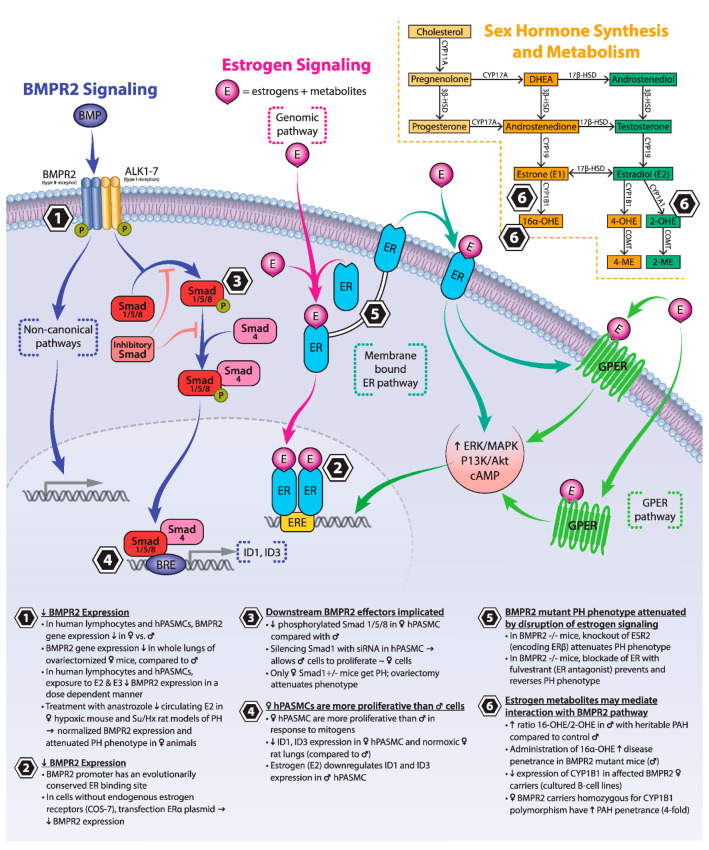
Overview of sex hormone synthesis and metabolism and the interaction between estrogen and *BMPR2* signaling pathways. BMP = bone morphogenetic protein; *BMPR2* = bone morphogenetic protein receptor 2; BRE = BMP response element; DHEA = dehydroepiandrosterone; E3 = estriol; ER = estrogen receptor; ERE = estrogen response element; GPER = G-protein-coupled estrogen receptor; hPASMC = human pulmonary artery smooth muscle cell; PH = pulmonary hypertension.

As discussed in the following subsections, multiple lines of evidence, in both health and disease, suggest that baseline BMPR2 expression and signaling may be reduced in females compared to males. A relative deficiency in BMPR2 expression in females may be the “second hit” required to reduce BMPR2 signaling below a critical threshold and allow disease penetrance in *BMPR2* mutation carriers (typically a haplo-insufficient state). However, interactions between estrogen and BMPR2 are complex and context-dependent, and may depend on such factors as patient age, menopausal status, cell type studied, and dose responses and time courses. Figure 1 summarizes key points of interaction between estrogen and *BMPR2* signaling.

### 5.1. Estrogens and Their Receptors Reduce BMPR2 Expression and Downstream Signaling

When assessed in human lymphocytes (commercial cell line) and pulmonary artery smooth muscle cells (hPASMCs; from healthy subjects; cell donor age range: 58–76 years), BMPR2 expression was shown to be lower in female cells compared to males [49,50]. BMPR2 expression in human lymphocytes is suppressed in a dose-dependent manner by the administration of both E2 and E3, with further suppression in the setting of proliferative signals [49]. Additionally, the *BMPR2* promoter has an active and evolutionarily conserved ER binding site [49]. Transfection of a cell line lacking endogenous estrogen receptors with increasing concentrations of ERα plasmid decreased activity at the *BMPR2* promoter (using a luciferase reporter construct), suggesting that direct binding of the ER to the *BMRP2* promoter site may be a mechanism for reduced BMPR2 expression in females [49].

Downstream effectors of BMPR2, including the phosphorylated-Smads 1/5/8 and ID1 and ID3, may also be reduced in female hPASMCs compared to males (healthy subject donors as described above) [50]. In keeping with this finding, female hPASMCs are more proliferative in response to mitogens compared to male cells (suspected to be due to reduced activity of the BMPR2 signaling pathway) and have reduced induction of phosphorylated-Smads when exposed to BMP4, an agonist of BMPR2. Silencing of *SMAD1* using microRNA, thus inhibiting the BMPR2 signaling pathway, allowed male hPASMCs to proliferate in a similar fashion to those of females [50].

Similar findings have been observed in murine models. When examined via whole-lung analysis in normal mice, *BMPR2* gene expression was lower in ovariectomized females compared to males [49]. These findings are corroborated by a second study which found that in normoxic rodents (mice and rats), lung transcript levels of *BMPR2* and downstream effectors ID1 and ID3 were significantly lower in females than males [51].

In two well-established experimental rodent models of PH (hypoxia (mouse) and sugen 5416 + hypoxia (rat; Su/Hx) [52]), Mair and colleagues demonstrated that BMPR2 levels were significantly downregulated in hypoxic male and female rodents compared to normoxic control animals. Treatment with the aromatase inhibitor anastrozole reduced circulating E2 levels in female rodents, with corresponding normalization of BMPR2 expression and attenuation of changes in right ventricular systolic pressure (RVSP) and pulmonary vascular remodeling [51].

### 5.2. Loss of Estrogen Signaling Attenuates Experimental PH Phenotypes Driven by Mutations in Components of the BMPR2 Signaling Pathway

Further evidence of the interplay between the *BMPR2* and estrogen signaling pathways comes from experimental PH models driven by mutations in *BMPR2* and *SMAD1*. Using anastrozole or the ER antagonist fulvestrant to inhibit estrogen signaling in *BMPR2*^−/−^ mutant mice, Chen et al. [53] demonstrated both prevention and reversal of the typical *BMPR2*-mutation-associated experimental PH phenotype. Knock-out of *ESR2* (the gene encoding estrogen receptor β), and less so *ESR1* (the gene encoding estrogen receptor α), reduced the elevation in RVSP typically seen in *BMPR2*^−/−^ mice, attenuated the muscularization of small pulmonary vessels, and eliminated the presence of vessel occlusion occasionally seen in *BMPR2*^−/−^ mutant mouse lungs [53].

Conditional knock-out of *SMAD1*, a receptor-regulated Smad phosphorylated by BMPR2, in either endothelial cells or smooth muscle cells of mice has been previously shown to cause elevated RVSP and increased muscularization of pulmonary arteries [54]. Interestingly, only female conditional knock-out *SMAD1*^+/−^ mice develop elevated RVSP and pulmonary vascular remodeling, and PASMCs isolated from these mice proliferated faster than those of female wild type mice, suggesting synergism between female sex and the heterozygous loss of *SMAD1* in adult mice. Ovariectomy attenuated the PH phenotype in *SMAD1*^+/−^ females, further suggesting that it is the presence of female sex hormones that drives a difference in penetrance between sexes [50].

### 5.3. Estrogen Metabolites May Mediate Interaction between BMPR2 and Estrogen Signaling

Recent attention to estrogen metabolites and related enzymes has provided more evidence supporting the interaction between *BMPR2* and estrogen signaling pathways in the development of PAH. CYP1B1 is a p450 enzyme that is highly expressed in lung tissue, and catalyzes 2- and 4-hydroxylation of estrogen. A different p450 enzyme catalyzes the hydroxylation of estrogen at C-16, primarily to 16α-hydroxyestrone (16α-OHE_1_). A low ratio of urinary 2-hydroxyestrogen (2-OHE)/16α-OHE_1_ is used as a biologic marker of decreased CYP1B1 activity. In contrast to 2- and 4-hydroxy estrogen metabolites (which are considered weakly or anti-mitogenic), 16α-OHE_1_ drives cellular proliferation via activation of the estrogen receptor, and preferential hydroxylation to 16α-OHE_1_ with a low 2-OHE/16α-OHE_1_ ratio has been associated with an increased risk of diseases resulting from the proliferative effects of estrogen signaling [55].

Comparison of gene array and RT-PCR from cultured B-cell lines of PAH-affected carriers of deleterious *BMPR2* variants, non-affected carriers, and normal control patients identified significantly lower expression of *CYP1B1* in the female PAH-affected *BMPR2* carriers [56]. A second study examined affected and unaffected female *BMPR2* mutation carriers for the presence of *CYP1B1 N453S*, a genetic polymorphism associated with increased protein degradation and previously implicated in hormone-related malignancies (breast, ovarian, prostate, and endometrial cancer) [57,58]. Similar to the results from West et al. [56], the investigation revealed a four-fold higher penetrance of PAH in carriers of deleterious *BMPR2* variants who were homozygous for the polymorphism. Supporting this genetic observation, the urinary 2-OHE/16α-OHE_1_ ratio was 2.3× lower in affected mutation carriers [58].

Two follow-up studies further examined the role of 16α-OHE_1_ in mediating the development of *BMPR2*-associated PH. Fessel et al. [59] found a higher ratio of 16α-OHE_1_/2-OHE (note that this is the inverse ratio from the previous study) in male patients with heritable PAH compared to healthy male controls, although the ratio was less divergent in males compared to the difference previously identified in females [59]. The same study demonstrated that in several genotypes of male *BMPR2* mutant mice (males were used to avoid the complexity of the native estrous cycle in female mice), administration of 16α-OHE_1_ roughly doubled disease penetrance (as defined by an elevated PVR) and, in one genotype, reduced cardiac output significantly. In a similar experiment, administration of 2-OHE was not found to be protective. Administration of 16α-OHE_1_ resulted in a relatively lung-specific decrease in Smad 1/5/8 phosphorylation and a decrease in BMPR2 protein level in control animals, but no further reduction in Smad phosphorylation in *BMPR2* mutants, suggesting a differential mechanism of how 16α-OHE_1_ may act as a second hit in the presence of *BMPR2* mutations. Gene expression data from these experiments demonstrated that in the presence of *BMPR2* mutations, 16α-OHE_1_ blunts classical cytokine and inflammatory signaling (as previously shown in the literature) but promotes vascular injury through unclear alternative mechanisms (angiogenesis, metabolism, and planar polarity). In a separate study, 16α-OHE_1_ was shown to upregulate the microRNA-29 family (miR-29) in *BMPR2* mutants, which alters energy metabolism; antagonism of miR-29 improved the in vivo and in vitro features of PH [60].

## 6. Conclusions

Taken together, scientific investigations demonstrate multifaceted interactions between estrogens, estrogen metabolites, and BMPR2 signaling. The data suggest that abnormalities in BMPR2 signaling pathways may synergize with estrogenic signaling to create a permissive environment for promoting PAH development. Such a paradigm could explain why there is a discrepancy between pro-proliferative and PAH-promoting estrogen effects noted in these studies and protective estrogen effects in other contexts and model systems [31]. In the context of genetic alterations in BMPR2 signaling, estrogens may exert PAH-promoting effects that they do not exert in other contexts. In particular, there may be a shift towards the production of mitogenic metabolites such as 16α-OHE_1_. Further study may elucidate exact mechanisms and potential therapeutic targets.
